# Cloze probability, predictability ratings, and computational estimates for 205 English sentences, aligned with existing EEG and reading time data

**DOI:** 10.3758/s13428-023-02261-8

**Published:** 2023-10-25

**Authors:** Andrea Gregor de Varda, Marco Marelli, Simona Amenta

**Affiliations:** Department of Psychology, University of Milano – Bicocca, Piazza dell’Ateneo Nuovo 1, Milano, MI 20126 Italy

**Keywords:** Cloze probability, Predictability ratings, Surprisal estimates, Prediction

## Abstract

We release a database of cloze probability values, predictability ratings, and computational estimates for a sample of 205 English sentences (1726 words), aligned with previously released word-by-word reading time data (both self-paced reading and eye-movement records; Frank et al., *Behavior Research Methods*, *45*(4), 1182–1190. 2013) and EEG responses (Frank et al., *Brain and Language*, *140*, 1–11. 2015). Our analyses show that predictability ratings are the best predictors of the EEG signal (N400, P600, LAN) self-paced reading times, and eye movement patterns, when spillover effects are taken into account. The computational estimates are particularly effective at explaining variance in the eye-tracking data without spillover. Cloze probability estimates have decent overall psychometric accuracy and are the best predictors of early fixation patterns (first fixation duration). Our results indicate that the choice of the best measurement of word predictability in context critically depends on the processing index being considered.

## Introduction

In recent years, the role of context-dependent probabilistic information in language processing has garnered significant attention from researchers in psycholinguistics and neurolinguistics. The general picture that is emerging from this research effort is that predictable words are processed faster (as shown by shorter gaze fixations, Ehrlich & Rayner, 1981; Staub, 2015; and self-paced reading times, Fernandez Monsalve et al., 2012; Frank & Hoeks, 2019) and elicit reduced neural signals associated with processing difficulty (N400 ERP component, DeLong et al., 2005; Kwon et al., 2017; Van Berkum et al., 2005; frontotemporal fMRI activation, Baumgaertner et al., 2002; Dien et al., 2008; and MEG responses, Takahashi et al., 2021). However, while now it is generally acknowledged that context-dependent probabilistic information plays a prominent role in language processing, there are still some major open questions regarding how the language processor makes use of these statistical data. It has been proposed that contextual word probabilities are employed to generate predictions about the upcoming lexical material (see for instance de Varda & Marelli, 2022; McDonald & Shillcock, 2003; Urbach et al., 2020), emphasizing the role of implicit expectations in language processing, in line with a “top-down” model of language comprehension (Lupyan & Clark, 2015). Nonetheless, there is still no consensus on the *linking function* that relates contextual word probabilities and cognitive effort, with various accounts proposing either a linear (Brothers & Kuperberg, 2020; Reichle et al., 2003), a logarithmic (Levy, 2008; Luke & Christianson, 2016; Smith & Levy, 2013; Shain et al., 2022), or a super-logarithmic relationship (Hoover et al., 2022; Meister et al., 2021). Furthermore, different accounts have proposed theoretically distinct reasons why predictive processing should take place in the first place, with some researchers proposing a main role in language learning (Chang et al., 2006) or facilitating mutual understanding (Pickering & Garrod, 2007), and others characterizing prediction in language understanding as a consequence of the general neural and functional organization of the human mind (Clark, 2013; see Huettig, 2015 for an overview). Crucially, these alternatives entail fundamentally different conclusions about the processes underlying the language system (Brothers & Kuperberg, 2020; Smith & Levy, 2013). A proper evaluation of these different perspectives is thus a central issue in confronting cognitive theories. The existence of linguistic stimuli with normed properties is of paramount importance to experiments aimed at contrasting theoretical accounts on the influence of context-dependent probabilistic information in language processing. Even when an experiment is not specifically aimed at investigating the effects of contextual predictability, the rigor of the conclusions that can be drawn from a sentence-level psycholinguistic study depends on accurately estimating stimulus predictability to include it as a statistical control.

The traditional approach to assess the contextual probability of a word $w_i$ consists in presenting participants with that word’s context (typically the previous words in the sentence, $w_{1}$, $w_{2}$ $\ldots$ $w_{i-1}$) and ask them to generate an appropriate continuation (Taylor, 1953). The proportion of people that picked the target word $w_i$ as the continuation of the sentence is referred to as the *cloze probability* of the word $w_i$, which is taken as an estimate of the subjective probability computed by a skilled reader during online comprehension^1^. Cloze probability has proven itself successful in predicting behavioral (Duffy et al., 1989; Luke & Christianson, 2016; Schuberth et al., 1981) and neural responses (DeLong et al., 2005; Kutas & Hillyard, 1984; Szewczyk & Federmeier, 2022). However, probabilistic data derived from the cloze test are expensive to collect, and they tend to provide unreliable estimates for low-probability words under realistic sample sizes (Shain et al., 2022). Note that this aspect is problematic not only from a methodological point of view but also from a theoretical one, since differences in processing cost associated with low-probability words are crucial in disentangling between the linear and the logarithmic accounts of the relationship between predictability and processing cost. As a way to offset this complication, researchers have sometimes employed *predictability ratings* from a normative group of participants, where the extent to which a word could be anticipated from the previous context is evaluated on a Likert scale (Brothers et al., 2020; DeLong et al., 2014; Rayner et al., 2001; Szewczyk & Federmeier, 2022)^2^. Predictability ratings have the advantage of providing predictability estimates for low-probability words, which would be rarely generated in a cloze task, if at all. However, both predictability ratings and cloze responses are collected as non-speeded, off-line measurements, which can be affected by conscious reflection and thus distorted by strategic effects (Kutas & Federmeier, 2011; Smith & Levy, 2011; Szewczyk & Federmeier, 2022). A third alternative to measuring contextual predictability relies on *computational estimates* obtained from large corpora of naturalistic text. These estimates are generally obtained from statistical language models developed in the field of natural language engineering. Statistical language models are usually trained to predict the next word in a sequence of natural text (a task that is referred to as causal or auto-regressive language modeling) and thus define a conditional probability distribution over the lexicon that can be employed as an estimate of word predictability in context. Computational estimates of word predictability have the undeniable advantage of generating probability distributions over the whole vocabulary, and thus are particularly suited to model the low-probability tail of the distribution. Computational estimates are an interesting option also from a methodological perspective, since they can account for human performance without requiring human annotation; indeed, it has been argued that it is preferable to devise non-self-referential explanations of human behavior starting from the objective properties of the stimuli (see for instance Günther et al., 2020; Günther et al., 2021; Westbury, 2016). However, it is still not clear whether computational estimates achieve the same psychometric predictive power that can be obtained by employing human annotation, since these data-driven probabilistic measurements have been shown to perform worse (Smith & Levy, 2011), on par (Shain et al., 2022) or even better (Hofmann et al., 2022; Michaelov et al., 2022) than cloze probability estimates obtained with human intervention. Furthermore, predictability estimates from causal language models fail to accurately account for the human processing difficulty of some specific linguistic constructions, such as garden path sentences (Arehalli et al. , 2022; Van Schijndel & Linzen , 2021), nested hierarchical structures (Hahn et al. , 2022), and grammatical violations (Wilcox et al. , 2021).

In light of the considerations reported above, it is clear that each of the three methodological options for the measurement of predictability has its own strengths and weaknesses. Each alternative might be more suited to certain research settings, depending on the focus and the theoretical motivation of the study. For instance, studies focusing on the influence of objective text-based statistical information in reading might be more prone to consider computational estimates. In contrast, studies that emphasize the role of subjective probabilistic knowledge might consider cloze probability or predictability ratings as better psychometric candidates. To promote a multifaceted approach to context-dependent sentence processing, we release a database of aligned cloze probability estimates, predictability ratings, and computational estimates for a sample of 205 sentences (1726 words) released by Frank et al. (2013).

Research in incremental language processing crucially relies on appropriate measurements of word predictability in context, i.e., on a proper operationalization of the independent variable that is assumed to influence anticipatory processing. However, in typical psycholinguistic studies, these variables are not considered by themselves, but need to be related to dependent variables reflecting the processing cost associated with word probability. For this reason, psycholinguistic resources aimed to foster research in sentence processing should ideally comprise both predictability estimates and empirical measurements of processing demands. In line with this rationale, the contextual predictability estimates that we release are aligned with previously released word-by-word reading time data (both self-paced reading and eye-movement records, Frank et al. (2013)) and EEG responses (Frank et al., 2015).

## Materials and methods

### Data

#### Cloze probability

Stimuli were extracted from the datasets by Frank et al. (2013) and Frank et al. (2015), for a total of 205 English sentences, ranging from 5 to 15 words. This initial sentence sample was used to obtain items for the cloze probability task: each sentence was progressively split at each word starting from the first one, so that for every sentence $n-1$ fragments were generated (where *n* is the length of the sentence in words). Table 1 reports an example of the items obtained from the sentence *The bored looking soldier just pointed*. Note that while in the table the sentence fragments are shown incrementally, during data collection they were presented in randomized order to the participants.

**Table 1:** Fragments and associated upcoming words obtained from the sentence “The bored looking soldier just pointed”, used as stimuli in the cloze probability and the predictability rating tasks

Sentence fragment	Upcoming word
The	Bored
The bored	Looking
The bored looking	Soldier
The bored looking soldier	Just
The bored looking soldier just	Pointed

Note that the associated upcoming word was only accessible to the participants in the second task

Following this procedure, a total of 1726 items were obtained. These were presented in a typical cloze-probability task: participants were asked to continue the sentence by writing what they expect to be the next word. The instructions stressed that, even if participants came up with several options, their task was always to produce one single word – the one that, in their immediate intuition, should follow what was presented. Moreover, it was emphasized that the to-be-produced word could belong to any part-of-speech, including articles and prepositions.

Data were collected in a series of crowdsourcing studies through Prolifc Academics (https://www.prolific.co/). The total item sample was randomly divided into 8 different lists, each including about 216 items. Each list was administered via a separate study, released via the crowdsourcing platform. A total of 80 participants were involved in each study, with a compensation of 5.63 pounds. All participants were self-declared English first-language speakers. As a further control, before the actual cloze probability task, participants were asked to complete a simple word knowledge test comprising ten items from Nation’s Vocabulary Size Test (Nation & Beglar , 2007). Participants that made more than two mistakes in the test were excluded. With this criterion, 13 participants were not considered in the following analyses. The median completion time was 27 min. The produced cloze responses were automatically spell-checked with the Python package pyspellchecker (version 0.7.1), a simple toolkit that combines frequency and Levenshtein orthographic distance to correct misspelled words. The records of all the corrections (3.95% of the produced words) are reported in the Supplementary Materials. Punctuation signs were not considered in the calculation of cloze probability estimates.

**Table 2:** Summary description of the computational models considered in this study

Model	Description
*n*-grams	*n*-grams are simply sequences of *n* words; an *n*-gram model estimates the probability of a word $w_i$ by only considering the previous $n - 1$ words - in other words, they approximate P($w_i$ | $w_1$, $w_2$, $\ldots$ $w_{i-1}$) as P($w_i$ | $w_{i-n+1}$ $\ldots$ $w_{i-1}$). This probability value is obtained by counting and normalizing the number of occurrences of word sequences of length *n*.
Phrase Structure Grammars	PSGs are models that take into account the hierarchical syntactic structures of the sentences (Chomsky , 1957). They are composed of a finite set of rules that governs the way some constituents (e.g., a Noun Phrase) may be composed by other constituents (an Adjective and a Noun), which in turn correspond to lexical items (*wooden*, *chair*). The PSG considered in this study is stochastic, and assigns a probability to each rule (Roark , 2001).
Recurrent Neural Networks	RNNs are neural networks that are trained to predict the next word given the previous sentence context. They are endowed with feedback connections that allow information to persist over time (Elman , 1990). Through these feedback loops they maintain an internal representation of the context that gets updated incrementally after each word. A detailed description of the RNN architecture employed in this study is presented by Fernandez Monsalve et al. (2012) and Frank (2013).
Transformers	Unlike RNNs, which process data sequentially, transformers process all the input data in parallel by using a mechanism called “self-attention” to weigh the importance of different parts of the input (Vaswani et al. , 2017). This allows transformers to better capture long-distance dependencies.

One common problem with cloze probability norms is that they often produce zero probability estimates for some items. In other words, there are some cases where the target word $w_i$ is not produced by any participant as a possible continuation of $w_1$, $w_2$ $\ldots$ $w_{i-1}$. This aspect is problematic both from a theoretical and a methodological point of view. First, it is unlikely that all the words that have not been produced in a cloze task correspond to impossible sentence continuations according to the participants’ subjective probability distribution; under realistic sample sizes, words with $p(w_i | w_1, w_2 \ldots w_{i-1}) < .001$ will be virtually absent from the participants’ responses. Intuitively, this problem highlights the need to divide the probability mass over more words than the ones that have been actually seen. Second, zero-probability events are problematic for the accounts that propose a logarithmic functional form of the effect of predictability on processing times since the logarithm of zero is undefined. As a way to offset these problems, we smoothed the probability distribution derived from cloze responses via Laplace smoothing (Eq. 1).

1 $$\begin{aligned} P_{smoothed}(w_i) = \frac{|w_i| + \alpha }{\sum ^{K}_{j = 1} (|w_j|) + \alpha \cdot K} \end{aligned}$$

Where $\alpha = 1$ is an additive constant, $|w_i|$ is the number of participants that produced $w_i$ in response to the cloze item, and *K* the size of the set of words produced in the cloze item.

As mentioned in the Introduction, the functional form of the effect of word predictability on processing cost is still a matter of debate. While there is ample consensus that probabilistic estimates extracted from statistical language models provide better fit indexes to behavioral data if log-transformed (see for instance Berzak & Levy, 2022; Shain et al., 2022; Smith & Levy, 2013; Wilcox et al., 2020), it has been argued that cloze probability values better predict human behavior if entered linearly in a statistical model (Brothers et al., 2020, but see Shain et al., 2022). In the following analyses, we chose not to commit *a priori* to a specific alternative, and considered both cloze probability (cloze_p_) and its negative log transformation (cloze_s_ $= - \log$ cloze_p_).^3^

#### Predictability ratings

The same items employed in the cloze-probability task (aggregated in the very same eight lists) were also administered in a rating experiment. In this case, participants were presented with both the sentence fragment and the associated upcoming word (see Table 1) and asked to rate, on a scale from 1 to 5, how much they would expect the presented word to follow the presented sentence fragment. Instructions emphasized that we were not asking to evaluate how plausible or sensible that word was, but rather how they expect to find it while reading the preceding sentence context.

Also in this case, data were collected in a series of eight crowdsourcing studies through Prolific Academics (https://www.prolific.co/). The same procedure described for the cloze probability task was followed. A total of 60 participants, who did not take part in the cloze data collection, were involved in each study, with a compensation of 3.13 pounds. Data from participants that made more than two mistakes in Nation’s Vocabulary Size Test were discarded. With this criterion, 14 participants were excluded. The median completion time was 22 min.

#### Computational estimates

Statistical language models vary by an ample margin in their architecture and computational complexity. Frank et al. (2013) have released, in conjunction with their ERP data, probabilistic estimates derived from several models (*N*-grams, Recurrent Neural Networks, and Phrase Structure Grammars, trained to predict both lexical items and Parts of Speech; see Table 2 for a brief description of the models). However, research in natural language engineering has made significant progress since 2015, mainly driven by the widespread adoption of transformer-based neural networks (Vaswani et al., 2017). Transformers are deep learning models that rely on the mechanism of self-attention, weighing the significance of each token in the input data in order to generate a prediction. They are designed to process sequential data in parallel and create probability distributions over the whole lexicon. Research in psycholinguistics has largely followed the progressive switch to the transformer architecture, with transformer-based predictability estimates being evaluated as indexes of processing difficulty (Wilcox et al., 2020; Hao et al., 2020; Merkx & Frank, 2021). In our dataset, we release probabilistic estimates derived from seven pre-trained transformer-based neural network models, with sizes ranging from 124 million to 2.7 billion parameters. Four models (GPT-2_124M_, GPT-2_355M_, GPT-2_774M_, GPT-2_1.5B_) belong to the GPT-2 family (Radford et al. , 2019), an array of auto-regressive language models; three models (GPT-Neo_125M_, GPT-Neo_1.3B_, GPT-Neo_2.7B_) are instances of the GPT-Neo class, an open-source alternative to GPT-2 and GPT-3 models trained on a more diverse sample of texts (Black et al. , 2021). In light of the ample evidence that computational predictability estimates have a logarithmic effect on processing times (e.g., Shain et al., 2022; Smith & Levy, 2013), we convert the word probability estimates extracted from the transformer models to surprisal values. In the case of multi-token words, we summed the log probabilities assigned to the sub-word tokens, following the chain rule.

### Employed variables

In this study, we considered a series of word predictability measurements (henceforth wpms) and neural and behavioral measures of processing demands (nbms).

We employed as wpms our measurements obtained via human annotation (rating, cloze_p_, cloze_s_) and our computational log-probability estimates (GPT-Neo_[125M, 2.7B]_, GPT-2_[124M, 1.5B]_). Besides our human-annotated measures and the outputs of the transformer models, we also considered the computational estimates released by Frank et al. (2015), namely the output of three *N*-gram models (bigram, trigram, tetragram), a Phrase Structure Grammar (PSG), and a Recurrent Neural Network (RNN). A summary with a short description of each model is reported in Table 2. Note that Frank et al. (2015) released several versions of the PSG and RNN, each trained on an increasingly large subsample of the available textual data. For simplicity, we only report the results of the models trained on all the available data. We also disregarded the surprisal estimates relative to parts of speech instead of lexical items.

The nbms that we considered are the self-paced reading times (SPR) and eye movement patterns (first fixation duration: FFix; first pass duration: FPass; go-past duration: GoPast; right-bounded time: RightBound) released by Frank et al. (2013); the ERP components we analyzed are the N400, (Early) Post-N400 Positivity (EPNP and PNP), (Early) Left Anterior Negativity (ELAN and LAN) and P600 components as released by Frank et al. (2015). The various nbms and the processing stages they are assumed to reflect are summarized in Table 3; for more details on the ERP time windows and electrode sites we redirect the reader to Frank et al. (2015); for detailed information on the eye-tracking records see Frank et al. (2013).

**Table 3:** Summary description of the neural and behavioral measurements considered in the study

Measure	Description
First fixation duration	The time spent on the first single fixation on $w_i$. It has been characterized as a measure of low-level orthographic and pre-lexical processes (Radach & Kennedy , 2013), early lexical access, and predictive processing (Demberg & Keller , 2008; Staub , 2015).
Gaze duration	The sum of the duration of fixations landing on $w_i$ before the gaze leaves it (i.e., the time spent looking at $w_i$ during the first pass of the gaze). This measure has been proposed as an index of the processing costs associated with lexical access, and possibly of early syntactic and semantic integration (Inhoff & Radach , 1998; Rayner , 1998).
Right-bounded time	Summed duration of all fixations on $w_i$ before the first fixation on a word further to the right; it thus includes gaze duration plus further fixations on $w_i$ after regressive eye movements.
Go-past time	Sum of the duration of all fixations from the time the gazes lands on $w_i$ up to the first fixation on a word further to the right. Note that this often includes not only fixations on $w_i$, but also fixations on words to its left. It is considered a high-level integrative measure, although it has been noted that the fact that it incorporates both the occurrence of a regression and re-reading of previous segments makes it a complex or even ambiguous eye-tracking measure (Radach & Kennedy , 2013).
Self-paced reading time	Participants press a key on a keyboard to control the pace at which they read; every time they press the button, they advance to the next word or phrase, and reading times are recorded. Self-paced reading times are influenced by semantic, syntactic (De Vincenzi et al. , 2003), and pragmatic factors (Ditman et al. , 2007).
N400	The N400 is a relative negativity with a centro-parietal distribution peaking around 400 ms after the presentation of $w_i$. Its amplitude is modulated by both low-level factors, such as frequency and orthographic and phonological factors, and high-level features that impact meaning processing (Kutas & Federmeier , 2011).
Early Post-N400 Positivity	An early ERP component with positive polarity arising around 500 ms after the presentation of $w_i$, particularly evident in prefrontal sites. It is thought to be modulated by purely lexical expectations, independently of the conceptual relationships between $w_i$ and the anticipated completion (Thornhill & Van Petten , 2012).
Post-N400 Positivity	The PNP is a positivity arising between 600 and 900 ms after the onset of $w_i$ ^a^. It has been functionally characterized in an analogous way as the EPNP (Thornhill & Van Petten , 2012).
Early Left Anterior Negativity	The ELAN component is a left anterior negativity peaking around 150 ms after the presentation of the word; it is considered to be indicative of fast and automatic first-pass parsing processes (Gunter et al. , 1999).
Left Anterior Negativity	The LAN component is a left frontal relative negativity observed around 350 ms after the onset of $w_i$ (Kaan & Swaab , 2003). It has been proposed as an index of phrase structure building processes (Friederici , 1995), although there have been proposals that link it to non-syntax-specific working memory processes (Kluender & Kutas , 1993).
P600	Late positive wave peaking about 600 ms after the onset of $w_i$ mainly in centro-parietal sites. This ERP component is thought to be indicative of syntactic reanalysis (Friederici et al. , 1996), late responses to semantic anomalies (Van Herten et al. , 2005), and compositional integration (Aurnhammer et al. , 2021).

^a^In the dataset released by Frank et al. (2015), however, the time window of interest for the PNP was reduced to 600–700 ms to minimize the effects of the upcoming word $w_{i+1}$ on the response to $w_i$

### Analyses

Following Frank et al. (2015), we discarded from our analyses words attached to a comma, clitics, and sentence-final words; after this exclusion, our analyses were carried out on *N* = 1487 words.

We first run descriptive analyses aimed at capturing the relationships between the measures we collected; following these, we report a series of inferential analyses to compare the predictive power of our measures in relation to several neural and behavioral indexes of processing difficulty.

We started by inspecting the Pearson correlations between all the available measures (i.e., both measures of predictability and processing), and complemented this analysis with a hierarchical clustering based on the correlation patterns. The clustering analysis was based on a dissimilarity matrix constructed as the negative absolute correlation matrix, and Ward’s method was employed as an agglomerative clustering criterion. The Ward’s method (Ward , 1963) finds at each step the pair of clusters that increases by the least amount the within-cluster variance after the merging is performed.

In a second step, we compared the predictive power of our predictability measurements and the ones released by Frank et al. (2015) in estimating word-by-word processing times (both during self-paced and natural reading) and ERP amplitudes. To perform such a comparison, we fit a series of linear^4^ regression models with each wpm $\times$ nbm combination as the independent and dependent variable, respectively. All the models included as covariates the position of the word $w_i$ in the sentence, subtitle-based log-frequency estimates (Brysbaert et al. , 2012), length (in characters), and all the two-way interactions between these covariates.

Note that self-paced reading times and, to a lesser extent, eye-movement patterns, are known to be sensitive to spillover effects (Frank et al. , 2013; Just et al. , 1982). In the context of information-theoretic approaches to psycholinguistics, a common procedure to capture these effects involves the inclusion of the values of the independent variables (frequency, length, surprisal) relative to $w_{i-1}$ and sometimes $w_{i-2}$ as covariates in the regression models (see for instance Berzak & Levy, 2022; Goodkind & Bicknell, 2018; Hao et al., 2020) or to consider as the interest area a region comprising the target and the following word (Smith & Levy , 2011). However, these analytical choices are problematic in the case of our study for three main reasons.

First, they limit the comparability of the results across measures, as eye-movement patterns are known to respond to properties of $w_{i-1}$, while self-paced reading times are considered as susceptible even to features of $w_{i-2}$ (see for instance Meister et al., 2022) and EEG data are not generally considered to be sensitive to spillover effects. Thus, controlling for spillover effects would imply analyzing the different nbms with different model specifications, limiting the extent of the comparisons that can be legitimately drawn.

Second, considering spillover effects produces a significant data loss, since the first *N* words (with *N* being proportional to the width of the assumed spillover effect) of each sentence have to be discarded as there are no previous words available for computing the relevant word properties. This problem is exacerbated by the fact that the discarded words always occur in the same sentence positions (i.e., the first words in each sentence), potentially altering in a systematic way the distribution of processing times and predictability estimates in the sample.

Third, with spillover effects it is more difficult to draw conclusions on the fine-grained temporal dynamics of language processing; indeed, if the processing cost on $w_{i}$ is explained on the basis of the properties of $w_{i-1}$ and $w_{i-2}$, all the processing indexes become closer to late measures, as they are modeled as a function of linguistic features that have been accessible to the language processor since it was processing the two preceding words.

On the other hand, spillover effects are documented in the literature, and by excluding them we do not account for a systematic source of variation in human responses.^5^ Furthermore, the spillover effects of surprisal differ in their extent across the different measurements considered; most of the slowdown associated with surprisal is localized on $w_i$ in the case of eye-tracking (Wilcox et al. , 2023; Smith & Levy , 2013), and on $w_{i+1}$ in the case of self-paced reading (Smith & Levy , 2013). This asymmetry entails that, by excluding spillover effects, the effect of surprisal on self-paced reading times might be specifically underestimated. Hence, we report here both the results obtained without considering the spillover, and the results obtained when accounting for spillover effects.

To evaluate the increase in the explained variance due to the inclusion of the wpm as a fixed effect, we compared each experimental model with a corresponding baseline model, which was identical except for the absence of the fixed effects of the wpm. The increase in explained variance was operationalized as the difference in the log-likelihood ($\Delta LogLik$) between the baseline and the experimental model, as common practice in computational psycholinguistics (Goodkind & Bicknell, 2018; Hao et al., 2020; Oh & Schuler, 2022; Kuribayashi et al., 2021). In assessing the best predictors of each nbm, we employed the Akaike information criterion (AIC; Akaike, 1998). We considered as best predictors (a) the ones associated with the highest log-likelihood (or, equivalently, the lowest AIC), and (b) with $\Delta AIC_i < 2$, where $\Delta AIC_i$ is the difference between the AIC of the considered model ($AIC_i$) and the lowest AIC among the alternative regression models ($AIC_{min}$; see Symonds & Moussalli, 2011; Richards, 2005).

In order to assess the robustness of the results of our inferential analyses, we further tested their generalizability with 5-fold cross-validation. Each of the folds was iteratively left out from the training data, and the regression coefficients were estimated on the remaining four folds; the left-out fold was then employed as a disjoint test set to assess the fit quality on unseen data. As in the previous analyses, fit quality was measured as the $\Delta LogLik$ in the left-out fold with respect to the baseline; *p* values were calculated with the likelihood-ratio test, and aggregated across folds with Fisher’s combined probability test.

The data and materials for all experiments are available at https://github.com/Andrea-de-Varda/prediction-resource.

**Fig. 1:**
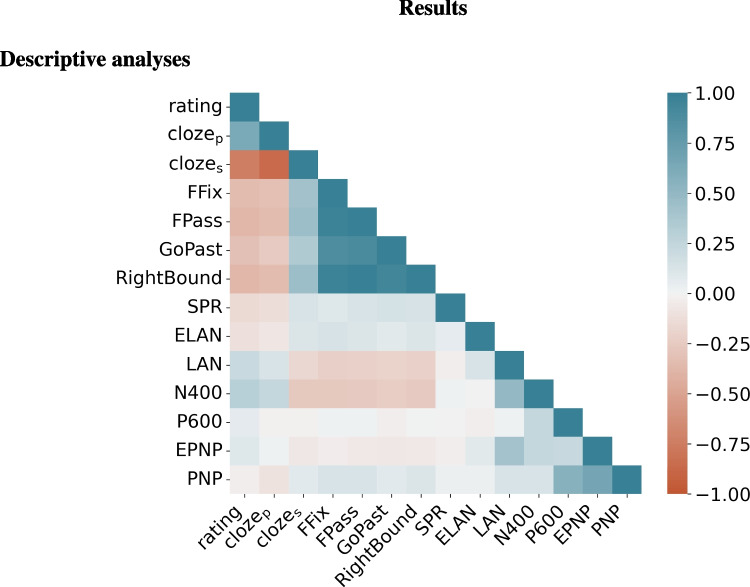
Correlation matrix including our measurements derived from human subjects (rating, cloze_p_, cloze_s_) and the various behavioral and neural indexes available for our dataset. To increase readability, the computational estimates have been excluded from the correlation matrix; the complete results are plotted in Appendix A and reported in detail in the online supplementary materials

**Fig. 2:**
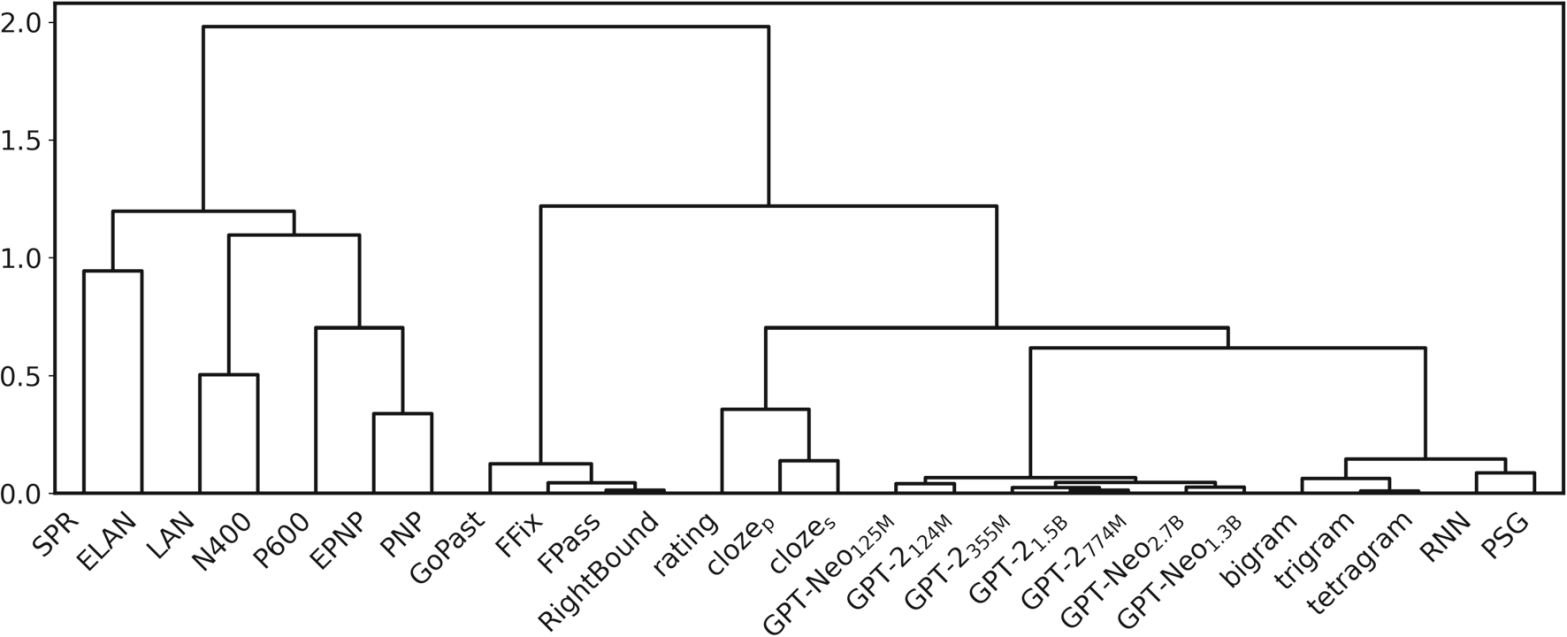
Hierarchical clustering of the computational, behavioral, and neural indexes available for our dataset

## Results

### Descriptive analyses

The correlation matrix including the human-derived wpms and all the nbms is reported in Fig. 1; the exact correlation coefficients are reported in detail in the online supplementary materials. The matrix shows that the lexical-predictability estimates (cloze_p_, cloze_s_, and ratings) are correlated; the sign of the correlations between cloze_p_ and rating on the one hand and cloze_s_ on the other hand is negative because of the conversion to surprisal. The eye movement patterns (FFix, FPass, GoPast, RightBound) are strongly related to each other, and moderately correlated with all the predictability measurements. Self-paced reading times do not display any notable preferential correlation with any predictability measurement; on the other hand, the amplitudes of the LAN, N400, and P600 ERP components show a positive relationship with predictability.

The results of our hierarchical clustering analysis are depicted as a dendrogram in Fig. 2. As can be seen in the figure, all the measurements we release display consistent correlational patterns, with our estimates based on human annotation (rating, cloze_p_, cloze_s_) and our transformer-based measures estimates (GPT-Neo_[125M, 2.7B]_, GPT-2_[124M, 1.5B]_) forming two clusters. Not surprisingly, our transformer-based predictability values are most strongly correlated with the text-based estimates released by Frank et al. (2015), i.e., *N*-grams, RNN, and PSG; then, the closest cluster is the one composed by our human-based estimates. The fact that human- and text-based estimates of lexical predictability are closer to each other than to the other variables corroborates the internal validity of our measures as indexes of context-dependent word predictability. On the above merging level, the predictability estimates are grouped with the eye-movement measures; this result shows that the indexes of processing difficulty that are more strongly correlated with word predictability in context are derived from gaze patterns. However, these correlational results should be taken with caution, since they do not partial out the effects of frequency, which is well known to correlate with both fixation durations (e.g., Carpenter & Just, 1983; White et al., 2018) and contextual probability^6^ (Ong & Kliegl , 2008; Moers et al. , 2017), resulting in possibly spurious correlations. The regression analyses described in the following subsection take into account this possible confound by including frequency as a covariate.

**Fig. 3:**
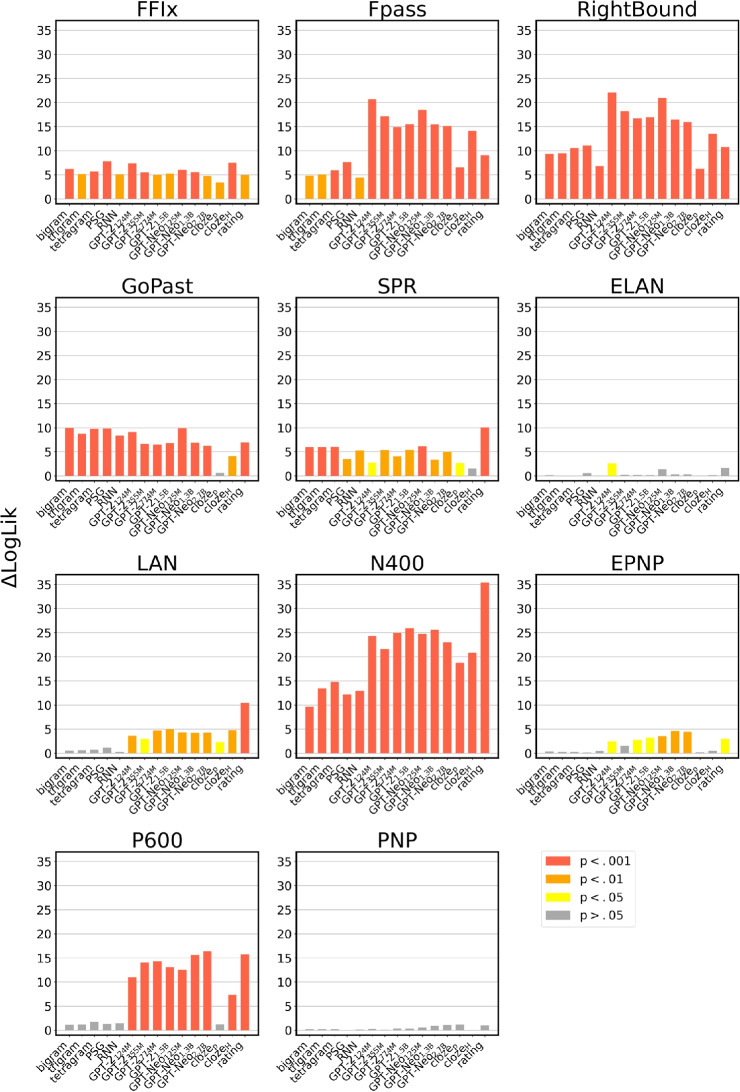
Increase in model fit ($\Delta LogLik$) in the linear regression models that could be ascribed to the inclusion of the wpms measuring predictability, with several neural and behavioral indexes of processing cost as dependent variables. The $\Delta LogLik$ values presented were obtained on the training data

### Inferential analyses

Figure 3 summarizes the results of 165 linear regression models (15 wpms $\times$ 11 nbms); more precisely, it plots the increase in model fit ($\Delta LogLik$) obtained by independently adding each measure of predictability as a fixed effect to the baseline model. The complete results are reported in Appendix B (Table 5). Our predictability measurements exert a reliable impact on various neural and behavioral processing indexes.

Most wpms are generally predictive of eye movement patterns. The earliest fixation measurement considered (FFix) is significantly associated with all the wpms; the most robust predictors are PSG ($\hat{B}$ = 1.8669, *t* = 3.9510, *p* = 0.0001, $\Delta LogLik$ = 6.1975), cloze_s_ ($\hat{B}$ = 2.3591, *t* = 3.8709, *p* = 0.0001, $\Delta LogLik$ = 7.4946) and GPT-2_124M_ ($\hat{B}$ = 1.3433, *t* = 3.8335, *p* = 0.0001, $\Delta LogLik$ = 7.3513).

In the case of first pass duration (Fpass), the best predictor is GPT-2_124M_ ($\hat{B}$ = 3.0184, *t* = 6.4610, *p* $< 0.0001$, $\Delta LogLik$ = 20.6923); from graphical inspection, Fpass results show a clear advantage of the transformer-based surprisal values over the computational predictability estimates released by Frank et al. (2015).

Then, the most robust predictor of RightBound is once again GPT-2_124M_ ($\hat{B}$ = 3.6020, *t* = 6.6772, *p* $< 0.0001$, $\Delta LogLik$ = 22.0787); like in the case of Fpass, the transformer-based estimates outperform simpler *N*-gram, PSG, and RNN models.

As for GoPast, several predictors satisfy the condition of being the best regressors in the analyses ($\Delta AIC_i < 2$); in particular, bigram ($\hat{B}$ = 5.0016, *t* = 4.4580, *p* $< 0.0001$, $\Delta LogLik$ = 9.9239), tetragram ($\hat{B}$ = 3.9031, *t* = 4.4178, *p* $< 0.0001$, $\Delta LogLik$ = 9.7471), PSG ($\hat{B}$ = 5.3099, *t* = 4.4360, *p* $< 0.0001$, $\Delta LogLik$ = 9.8268), GPT-2_124M_ ($\hat{B}$ = 3.7861, *t* = 4.2642, *p* $< 0.0001$, $\Delta LogLik$ = 9.0851), and GPT-Neo_125M_ ($\hat{B}$ = 3.8111, *t* = 4.4487, *p* $< 0.0001$, $\Delta LogLik$ = 9.883) have a comparable performance in accounting for GoPast reading times. Taken together, the results on eye-movement data show that fixation patterns are generally best accounted for by text-based measurements (and in particular transformer-based surprisal values) rather than human-derived estimates. One notable exception is FFix, which counts cloze_s_ as one of its best predictors.

SPR times are best predicted by the ratings ($\hat{B}$ = -4.1687, *t* = -4.4909, *p* $< 0.0001$, $\Delta LogLik$ = 10.07); the simpler measurements released by Frank et al. (2015) and the transformer-based computational estimates obtain comparable results; for instance, GPT-Neo_125M_ and tetragram are virtually indistinguishable from a model selection perspective ($\Delta AIC = 0.1659$).

In describing the results obtained with the ERP data, we will focus on the LAN, N400, EPNP, and P600 components. Indeed, no significant effect was found in PNP, and only one wpm reached statistical significance with ELAN amplitudes as dependent variables, without surviving to cross-validation (see the following section).

The strongest predictor of LAN amplitudes are the ratings ($\hat{B}$ = 0.2018, *t* = 4.5639, *p* $< .0001$, $\Delta LogLik$ = 10.3975); the other significant regressors are the cloze-based measurements and all the transformer-based estimates.

The N400 component is then the nbm for which our predictability estimates provide the starker increase in model fit. Its amplitude is best predicted by the ratings ($\hat{B}$ = 0.3084, *t* = 8.4921, *p* $< .0001$, $\Delta LogLik$ = 35.3873).

Nonetheless, all the wpm we consider produce a considerable increase in explained variance. As for the EPNP ERP component, our results show that its amplitude is better predicted by the GPT-Neo family, and in particular the two largest models (GPT-Neo_1.3B_: $\hat{B}$ = -0.0243, *t* = -3.0294, *p* = 0.0025, $\Delta LogLik$ = 4.5995; GPT-Neo_2.7B_: $\hat{B}$ = -0.0251, *t* = -2.9765, *p* = 0.003, $\Delta LogLik$ = 4.4407). EPNP is the only ERP measure that is not better accounted for by the ratings; the other regressors that are significantly associated with its amplitude are all the transformer-based models but GPT-2_355M_.

The last component we consider is the P600; the regressors associated with the most considerable increase in model fit are the ratings ($\hat{B}$ = 0.1847, *t* = 5.6200, *p* $< 0.0001$, $\Delta LogLik$ = 15.7095), GPT-Neo_2.7B_ ($\hat{B}$ = -0.0513, *t* = -5.7444, *p* $< 0.0001$, $\Delta LogLik$ = 16.4045), and GPT-Neo_1.3B_ ($\hat{B}$ = -0.0478, *t* = -5.5979, *p* $< 0.0001$, $\Delta LogLik$ = 15.5871). All the transformer-based models are predictive of the P600 amplitude, with relatively high $\Delta LogLik$ values; the models with more parameters (e.g., GPT-Neo_2.7B_, GPT-Neo_1.3B_) tend to outperform their under-parametrized counterparts. Note that this trend, which we also found in the case of EPNP, is in contrast with what we reported with eye-movement data, where smaller transformer models consistently outperformed the largest GPT-2 and GPT-Neo variants. Overall, a notable pattern that characterizes the ERP data is that, with the exception of the N400, EEG measurements are not significantly associated with the predictability estimates obtained by the simpler statistical models released by Frank et al. (2015); indeed, predictability effects in LAN, EPNP, and P600 only emerge when employing predictability ratings, transformer-based surprisal values, and, in the case of P600 and LAN, cloze_s_.

To summarize our results, we report in Table 4 an outline of the nbms that are best fitted by the regressors of interest. The table clearly shows that the ratings are particularly effective in the prediction of self-paced reading times and EEG responses, while GPT-2_124M_ is particularly suited at capturing eye movement patterns. Overall, transformer-based measurements tend to outperform all the *N*-gram models, PSG, and RNN.

**Table 4:** The last column of the table reports the nbms that are best fitted by the wpms considered in this study

Measure	Best predictor of
Bigram	GoPast
Trigram	None
Tetragram	GoPast
PSG	FFix, GoPast
RNN	None
GPT-2_124M_	FFix, Fpass, RightBound, GoPast
GPT-2_355M_	None
GPT-2_774M_	None
GPT-2_1.5B_	None
GPT-Neo_125M_	GoPast
GPT-Neo_1.3B_	EPNP, P600
GPT-Neo_2.7B_	EPNP, P600
Cloze_p_	None
Cloze_s_	FFix
Rating	SPR, LAN, N400, P600

**Fig. 4:**
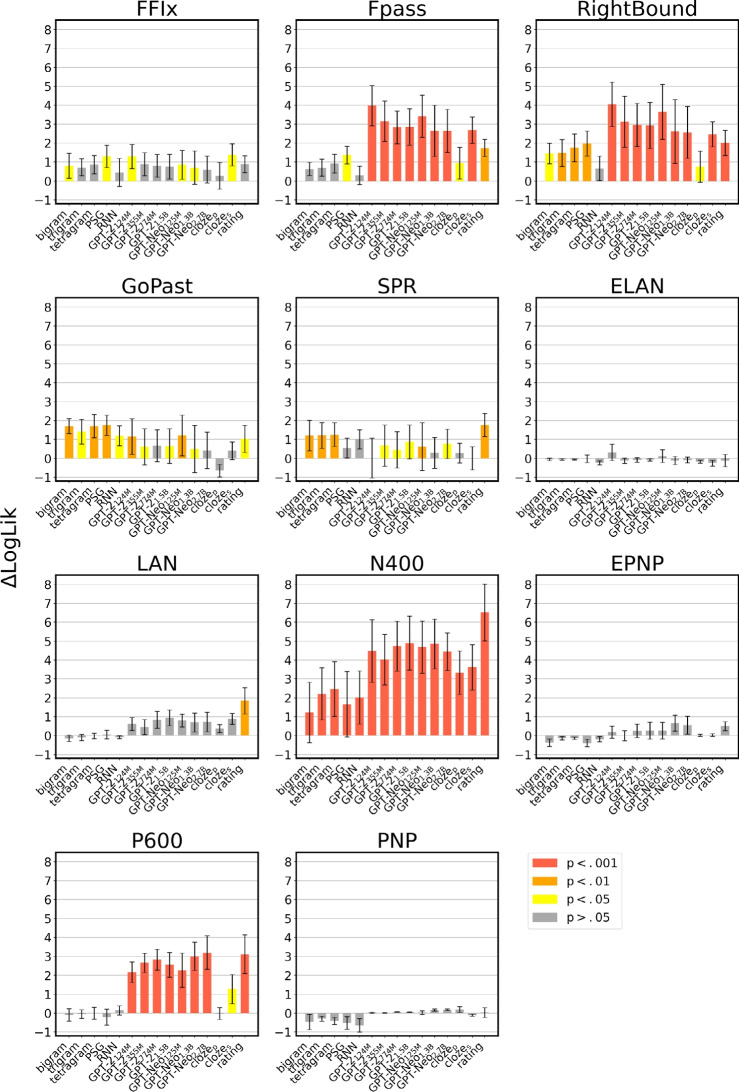
Results of the cross-validation analyses. The increase in model fit ($\Delta LogLik$) obtained in the linear regression models is averaged over the fivefold cross-validation. The error bars indicate the standard error of the mean

### Cross-validation

The robustness of the results we obtained in the previous section was assessed by means of 5-fold cross-validation. The results of the analyses are depicted in Fig. 4, and reported in detail in the online supplementary materials. Overall, cross-validation confirmed the results of our previous analyses. While the $\Delta LogLik$ are consistently lower (as expected, since the models are trained and tested on different folds of the data, with a more conservative approach), the patterns of results are very similar with respect to what we reported in the previous section; indeed, the average rank correlation between the results obtained in the previous section and the results of the cross-validation analyses is $\rho = 0.8568$.

In the case of FFix, Fpass, and RightBound, the best predictors are the same that have been previously identified. In the case of GoPast, bigram, tetragram, and PSG still achieve the best predictive power (bigram: $\Delta LogLik$ = 1.7007, SE = 0.4018, *p* = 0.0026; tetragram: $\Delta LogLik$ = 1.6998, SE = 0.6167, *p* = 0.0034; PSG: $\Delta LogLik$ = 1.7447, SE = 0.5334, *p* = 0.0027), while GPT-2_124M_ ($\Delta LogLik$ = 1.1544, SE = 0.9373, *p* = 0.0089) and GPT-Neo_125M_ ($\Delta LogLik$ = 1.2034, SE = 1.0754, *p* = 0.0023) perform generally worse in the cross-validation setting. SPR times are once again best predicted by the ratings ($\Delta LogLik$ = 1.7591, SE = 0.6037, *p* = 0.0029); however, GPT-Neo_125M_ ($\Delta LogLik$ = 0.6128, SE = 1.2656, *p* = 0.0057) underperforms in the cross-validation settings.

The most notable differences with the results described in the previous section are found in the ERP data. Effects on EPNP amplitudes, which were previously found to be associated with several nbms, did not survive the cross-validation analyses. Similarly, none of the previously significant predictors of LAN responses were sufficiently robust to hold in a cross-validation setting, with the exception of the ratings ($\Delta LogLik$ = 1.8395, SE = 0.6936, *p* = 0.0013).^7^ The pattern of results when considering N400 and P600 amplitudes was virtually unchanged after cross-validation, as the correlation with the previous results is (near-)perfect (N400: $\rho = 1$; P600: $\rho = 0.975$).

**Fig. 5:**
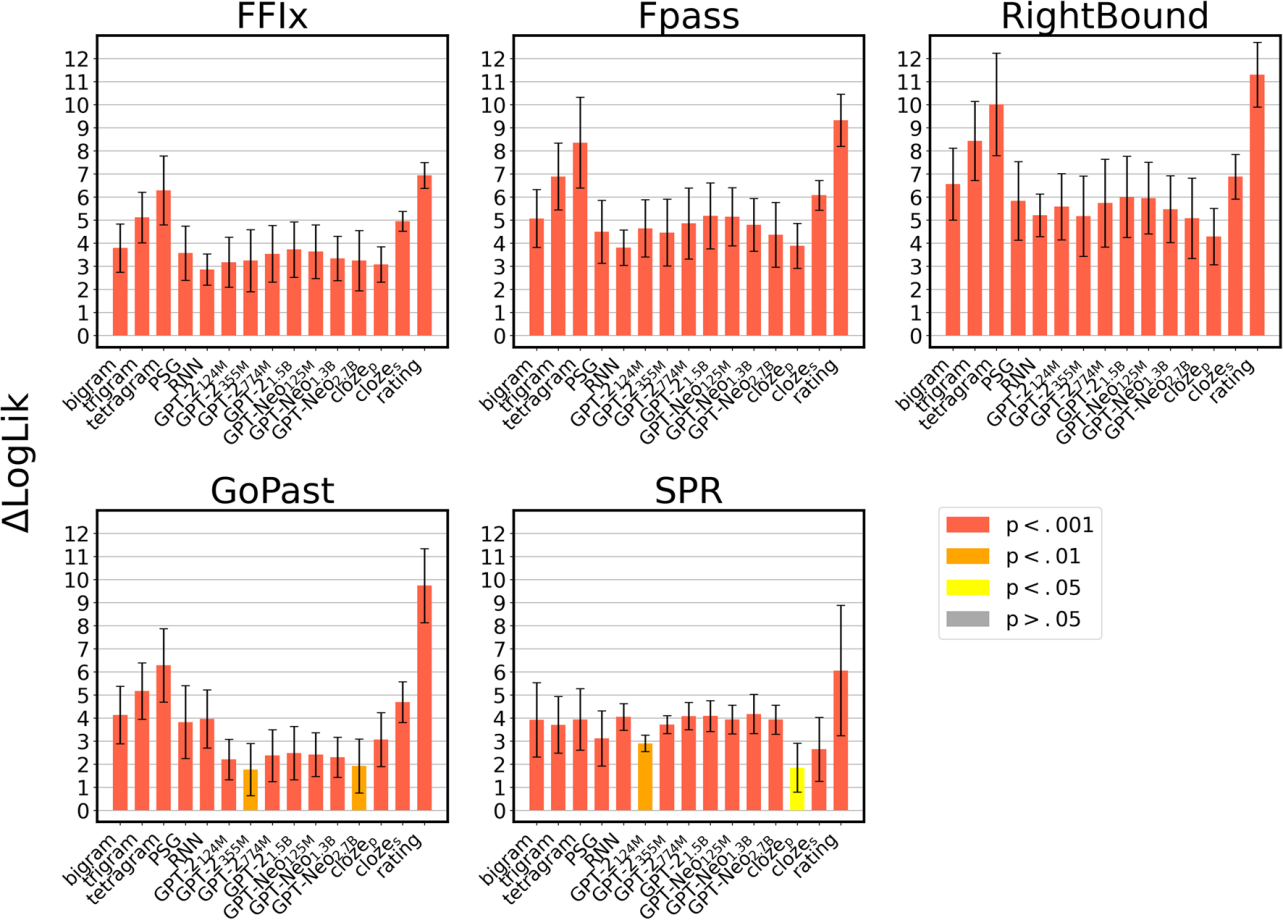
Effects of predictability on eye movement and self-paced reading data, with spillover

### Spillover effects

Self-paced reading times and fixation patterns are known to be sensitive to properties of the previous words. In the main results section, we decided to model reading times as a function of the properties of $w_i$, in order to (i) increase the comparability of our results across measures, (ii) limit the data loss, and (iii) compare wpms across early and late processing measurements (see the Analyses section). Nonetheless, for the sake of completeness and to better capture the specificity of each measurement, we report in this section the results of our spillover analyses.

In this section, we analyzed fixation times on $w_i$ as a function of the properties of $w_{i}$ and $w_{i-1}$, and self-paced reading times as a function of the properties of $w_{i}$, $w_{i-1}$ and $w_{i-2}$; our testing procedure was identical to the cross-validation analyses with respect to model configuration and considered variables. Note that the spillover analyses were carried out on *N* = 1090 words (as opposed to 1487 words without spillover), hence these results are not directly comparable to those of previous analyses.

The results of the spillover analyses are depicted in Fig. 5, and reported in the online supplementary materials. The figure shows that, numerically, predictability ratings display an advantage over the other wpms in explaining both fixation patterns and self-paced reading times. The other wpms that satisfy the criteria of being the best predictors for each measurement are cloze_s_ ($\Delta LogLik$ = 4.9528, SE = 0.4316, *p* < 0.0001), tetragram ($\Delta LogLik$ = 6.2884, SE = 1.4978, *p* < 0.0001), and trigram ($\Delta LogLik$ = 5.1188, SE = 1.0968, *p* < 0.0001) in the case of FFix; tetragram in the case of Fpass ($\Delta LogLik$ = 8.3601, SE = 1.9676, *p* < 0.0001) and RightBound ($\Delta LogLik$ = 10.0153, SE = 2.2233, *p* < 0.0001); and GPT-Neo_1.3B_ ($\Delta LogLik$ = 4.1723, SE = 0.8479, *p* < 0.0001), GPT-2_774M_ ($\Delta LogLik$ = 4.0821, SE = 0.5914, *p* < 0.0001) and GPT-2_1.5B_ ($\Delta LogLik$ = 4.0850, SE = 0.6649, *p* < 0.0001) for self-paced reading times.

As we argued in the Methods section, if the processing cost on $w_i$ is explained on the basis of properties relative to the previous words, even early processing measures such as FFix and Fpass become closer to late measures, since they are characterized as a cognitive response to information that was available to the language processing system when it was processing $w_{i-1}$. We propose that the edge that predictability ratings show over the other measurements could be interpreted under this account; when reading patterns are accounted for by the properties of the previous words, non-speeded responses based on conscious reflection provide the best wpms. We further note that accounting for spillover effects increased the relative predictivity of the *N*-gram models.

Note that, when accounting for spillover effects, all the eye movement processing indexes display similar patterns of results, as all the predictors achieve similar psychometric predictive power across measurements (average rank correlation $\rho$ = 0.8268, as opposed to $\rho$ = 0.3012 in the linear analyses and $\rho$ = 0.2482 with cross-validation). This observation corroborates our choice to separately analyze spillover effects and the effects localized on $w_i$ to better exploit the information provided by the different eye movement measurements.

## Discussion

Our results show that, overall, predictability ratings obtain very high psychometric accuracy, outperforming the other predictors in explaining self-paced reading, eye-tracking (with spillover), and EEG data. Predictability ratings are far less considered than cloze probability and computational estimates in the literature on prediction in incremental sentence processing (see for instance Hofmann et al., 2022; Merkx & Frank, 2021; Michaelov et al., 2022); our findings however suggest that the speakers’ explicit judgments on the predictability of a word given the previous context are highly predictive proxies of the processing cost associated with that word. On the other hand, cloze probability lags behind both ratings and modern transformer-based surprisal estimates. This finding corroborates the proposal that some data-driven probabilistic measurements can perform better than cloze probability estimates obtained with human annotation (Hofmann et al., 2022; Michaelov et al., 2022). However, we also show that, with appropriate sample size and some design choices such as a suitable smoothing technique, log-transformed cloze probability estimates are highly accurate predictors of *early* eye-movement patterns. In particular, we show that cloze_s_ is one of the best estimates in explaining the variance of FFix, both with and without spillover.

Notably, cloze probability is consistently associated with higher $\Delta LogLik$ values if entered logarithmically as a regressor; indeed, cloze_s_ is a better predictor than cloze_p_ in all the significant regression models (except in the case of SPR without spillover). This difference provides empirical support to the inferential theories of language comprehension, which advocate that the functional form of the effect of word predictability on cognitive effort should be indeed logarithmic (Levy, 2008; Luke & Christianson, 2016; Smith & Levy, 2013; Shain et al., 2022). Brothers & Kuperberg (2020) suggested that a logarithmic linking function between predictability and processing cost might be a spurious finding resulting from the employment of computational estimates of word probability in context instead of subjective measurements; our results, however, show that the result holds when employing a measure of subjective probability as the independent variable (see also Shain et al., 2022).

Another clear pattern that emerges from our results is that, among the text-based estimates and the sizes tested – which are not balanced across models –, transformers have an edge over *N*-grams, PSG, and RNN. Among the transformer-based models, however, the strongest predictive performance is obtained by GPT-2_124M_, which is the smallest model of the GPT-2 family and is among the best predictors of four different eye-tracking measurements (FFix, Fpass, RightBound, GoPast without spillover). Within each model family, smaller models tend to show a slight advantage over their over-parametrized counterparts, at least when considering eye-movement data.

This result supports the recent finding that larger pre-trained transformers, which generally obtain better results in next-word prediction and various downstream tasks, provide worse psychometric estimates than their smaller analogs (Oh et al. , 2022; Shain et al. , 2022; de Varda & Marelli , 2023).^8^ It has been proposed that this “inverse scaling” trend is due to the tendency of larger models to memorize word sequences during training, which causes their expectations to diverge from the ones that humans deploy during online sentence processing (Oh & Schuler , 2022). This pattern is consistent when considering eye-movement-based measurements of processing difficulty; with the ERP data, however, the comparison between the different transformer architectures is less straightforward, and in the case of the P600 and EPNP components bigger models have actually higher psychometric predictive power.

The trends we described above highlight the fact that some models are generally better at explaining variance in neural and behavioral indexes of processing cost. However, a critical aspect of our results is that not all processing measurements are best modeled by the same regressors. For instance, in the case of FFix,^9^ which is the earliest fixation measurement considered, simpler text-based estimates such as the PSG perform on par or even better than the deep transformer-based surprisal values. Similarly, also GoPast reading times are well described by both relatively simple models such as the *N*-grams and deep transformers with hundreds of millions of parameters (although the *N*-gram models are more robust to cross-validation). On the other hand, there is a stark difference in the predictive power of the transformer-based estimates and the statistical predictability measures released by Frank et al. (2015) when considering Fpass and RightBound, where the former measurements display a clear advantage. We propose that this asymmetry in the explanatory power of the computational predictability estimates might arise from the computational complexity of the cognitive processes being modeled. FFix is a very early eye-tracking measurement that is assumed to be indicative of low-level oculomotor processes, early lexical access, and predictive processing (Demberg & Keller , 2008; Staub , 2015); Fpass is thought to reflect lexical access and early semantic and syntactic integration (Inhoff & Radach , 1998; Rayner , 1998), and while we are not aware of an accepted functional characterization of RightBound, the measure subsumes both FFix and Fpass while including later fixations on $w_i$, arguably incorporating informative data on subsequent processing stages (see Table 3). GoPast has been described as an ambiguous measurement (Clifton et al. , 2007), as it incorporates the occurrence of a regression, indicating an arguably early difficulty in integrating a word, and also the cost of overcoming this difficulty through re-reading, which may reflect a late processing effect.^10^ Thus, relatively simpler computational models excel at explaining early processing while deep neural architectures are better at accounting for variance in intermediate-to-late integrative operations; hybrid measurements such as GoPast are then well modeled by both kinds of predictability estimates. When spillover effects are taken into account, even early measurements such as FFix are modeled as responses to the surprisal of the previous word, thus partially losing their status of early measurements. Thus, it is not surprising to find predictability ratings (non-speeded responses based on deliberate reflection) among the best predictors of all eye movement measurements.

SPR data are best fitted by predictability ratings, both with and without spillover; simple (e.g., tetragram) and deep transformer-based computational estimates (e.g., GPT-Neo_125M_) obtain comparable results (although also in this case the *N*-gram models are more solid in the cross-validation analyses), and cloze probability reaches statistical significance only if entered linearly in the regression model. The fact that ratings show the most substantial effect on SPR times is consistent with the observation that SPR is a consciously controlled method of progressing through a sentence, which puts within the reader’s intentional influence the criteria to employ for pressing the spacebar (Clifton & Staub , 2011). Analogously, predictability ratings are an explicit measure produced after conscious reflection; hence, the fact that they are the best predictors of SPR might be motivated by the fact that both kinds of behavior are the product of deliberate processes, possibly subjected to strategic effects. However, as for all the late processing measurements, SPR times also inevitably incorporate earlier components of the reading behavior, which may explain why also the simpler statistical estimates achieve a decent psychometric power. That said, we stress that these results should be interpreted with caution, as SPR can yield noisy data (Boyce et al. , 2020).

The idea that different operationalizations of the predictability construct better capture different processing indexes is well exemplified by the EEG data considered in this study.^11^ The LAN component is associated with our human-derived estimates and the transformer-based computational measures; the fact that the most complex predictability estimates explain such an early measurement might seem anomalous, but Frank et al. (2015) computed the LAN amplitude on a time window that was partially overlapping with the N400 (LAN: 300–400 ms; N400: 300–500 ms), and on adjacent electrode sites. This observation, in conjunction with the relatively high correlation between the recorded amplitudes of the two components (*r* = 0.4967, *p* $< .0001$), suggests that the results obtained with the former might be spurious correlations. We additionally note that LAN effects were robust to cross-validation only if entered non-linearly as predictors in the regression (see Appendix C). In the context of this article, we refrain from interpreting the results we obtained with the EPNP component, as no wpm is predictive of its amplitude in the cross-validation setting, even if modeled as a non-linear spline.

The N400 ERP component, in contrast, is significantly associated with all the predictability measurements considered; its stronger regressors are the predictability ratings, but all the other wpms are robust after cross-validation. The fact that the N400 component is well predicted by both shallow and deep statistical information as well as human-derived estimates is not surprising, given the vast array of information that the N400 has been shown to respond to. Indeed, it has been demonstrated that its amplitude is modulated by expectations driven by text-based distributional information (Frank et al. , 2015), orthographic (Laszlo & Federmeier , 2009), semantic (Kutas & Federmeier , 2000), and pragmatic factors (Van Berkum et al. , 2009), and it is also susceptible to violations related to broad world knowledge (Hagoort et al. , 2004). Given the wide range of information sources that inform the predictions the N400 is susceptible to, it is not surprising that all the operationalizations of predictability that we consider concur in explaining its amplitude.

The P600 component, on the other hand, is associated only with cloze_s_, ratings, and the transformer-based estimates, while the more shallow predictions generated by the simpler computational models do not achieve statistical significance. Its best regressors are the ratings GPT-Neo_2.7B_, and GPT-Neo_1.3B_, which are the biggest models considered in this study. These outcomes are in line with the functional role that has been proposed for the P600. This ERP component is an EEG signature that peaks 200 ms after the N400, and thus, from a purely temporal perspective, it is a later processing measurement with respect to the N400. The P600 amplitude has been shown to be modulated by late, controlled syntactic reanalysis or repair (Friederici et al. , 1996), late meaning-related responses to a sentence elicited after some semantic anomalies are detected (Van Herten et al. , 2005), and compositional integration in general (Aurnhammer et al., 2021; see Table 3). Given that the P600 occurs in a later processing stage than the N400 and is assumed to be indicative of complex integrative operations, the fact that its amplitude is predicted by large context-aware networks and non-speeded human responses but not by simpler computational models does not come as a surprise. This pattern, along with the eye-tracking results described above, is in line with the observation that it is not appropriate to adopt a “one-size-fits-all” approach when studying the role of linguistic statistical information across different cognitive processes (Wingfield & Connell , 2022), and speaks in favor of a flexible approach in choosing the appropriate computational estimate in cognitive modeling and psycholinguistics, which should take into account the complexity of the process being studied.

## Conclusion

In this study, we presented a set of data-driven and human-derived variables operationalizing the predictability of a word in context, and compared their psychometric predictive power in explaining the variance of several indexes of processing demands. Our results showed that, overall, predictability ratings are among the best predictors of cognitive cost during online sentence comprehension, showing a particularly marked advantage over the other measures in predicting SPR times, ERP responses, and eye-tracking data when spillover effects are accounted for.

Transformer-based surprisal estimates, on the other hand, excel in accounting for eye movement data without spillover effects. Lastly, log-transformed cloze probability estimates have decent average psychometric accuracy and are the best regressors of early eye-movement data (FFix); nonetheless, they lag behind the other two alternatives in all the other measures of processing cost. Taken together, our results highlight the importance of choosing the appropriate predictability measurement in cognitive research, which crucially depends on the processing index being considered.

We believe that the measurements we release can foster cognitive research in different ways. For instance, our measurements of predictability can serve as independent variables to aid the study of the interplay between sentence-level and word-specific properties in language comprehension (see for instance Amenta et al., 2022; Dambacher et al., 2006). Furthermore, we consider that our human-derived measurements constitute a rich and interesting behavioral phenomenon *per se*: in accordance with the dominant approach in the literature, we considered predictability ratings and cloze responses as independent variables to account for implicit indexes of processing cost; however, from a different standpoint, one might examine them as dependent variables that need to be explained on the basis of their objective properties. Within such an approach, one may take into account the whole distribution of responses for each item – a kind of information that is inevitably lost when considering word predictability as a single, word-level scalar as we did in this study. For instance, when calculating the cloze probability of $w_i$, one only considers the ratio of participants that produced $w_i$, ignoring other potentially relevant properties of the response distribution. Indeed, recent studies are starting to focus on the variability in the individual responses in several domains such as object naming (Gualdoni et al. , 2022), interpretation of compound words (Guenther & Marelli , 2022), and cloze distributions (Eisape et al. , 2020). We believe that predictability ratings and cloze responses could constitute a valuable tool to investigate the processes underlying both evaluative and productive components in sentence processing.

Our release of ratings and cloze responses contributes to a growing database of psychometric data collected on the same set of sentence stimuli, which for now covers two behavioral paradigms (eye-tracking and self-paced reading), EEG data, and several predictability norms. Future norming studies might expand the dataset with other behavioral measures of incremental language comprehension difficulty, including for instance Maze data.

## Figures and Tables

**Fig. 6:**
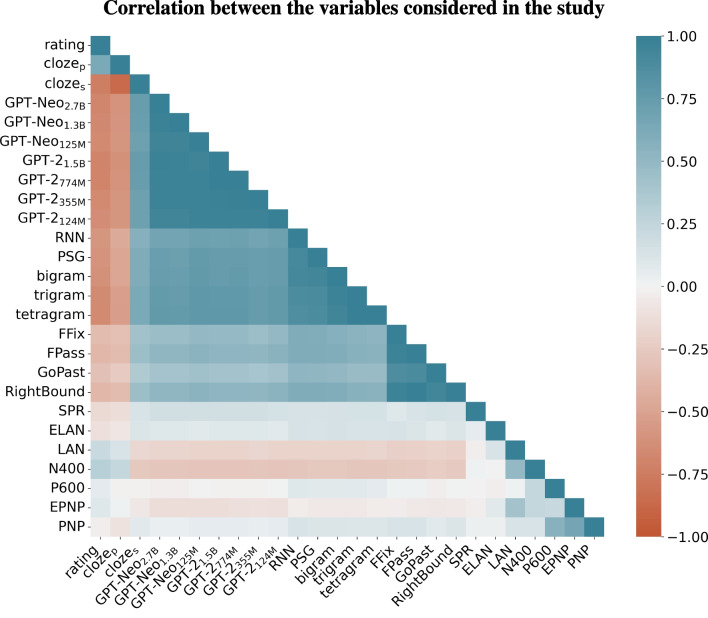
Correlation matrix including human-derived predictability measurements, transformer-based computational estimates (GPT-Neo_[125M, 2.7B]_, GPT-2_[124M, 1.5B]_), and the various behavioral and neural indexes of cognitive cost

**Fig. 7:**
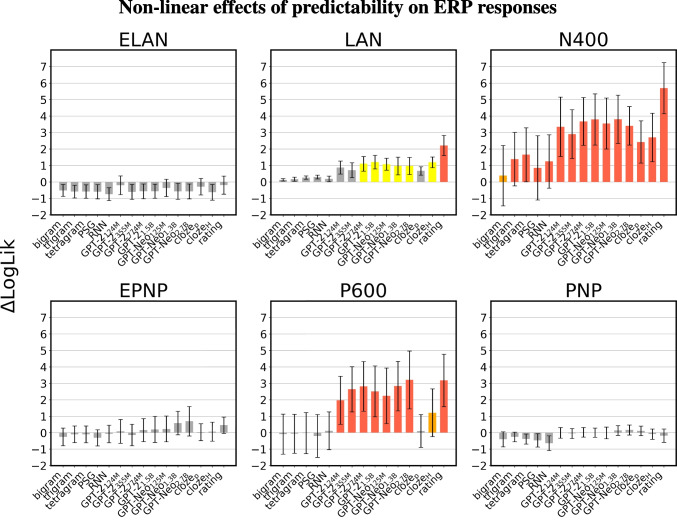
Non-linear effects of predictability on EEG data. The increase in model fit ($\Delta$ *LogLik*) obtained by adding a spline term for the wpms to the GAMs is averaged over the fivefold cross-validation. The *error bars* indicate the standard error of the mean

## Appendix B: Detailed results of the regression analyses

**Table 5:** Detailed results of the regression analyses

IV	DV	$\hat{B}$	*t*	*p*	$LogLik_{model}$	$LogLik_{baseline}$	$\Delta LogLik$	$\Delta AIC$
bigram	FFIx	1.5601	3.5185	0.0004	-7059.4158	-7065.6134	6.1975	-10.3951
trigram	FFIx	1.1939	3.2132	0.0013	-7060.4409	-7065.6134	5.1725	-8.3450
tetragram	FFIx	1.1731	3.3591	0.0008	-7059.9626	-7065.6134	5.6508	-9.3016
PSG	FFIx	1.8669	3.9510	0.0001	-7057.8069	-7065.6134	7.8065	-13.6129
RNN	FFIx	1.5795	3.1949	0.0014	-7060.4995	-7065.6134	5.1139	-8.2278
GPT-2_124M_	FFIx	1.3433	3.8335	0.0001	-7058.2621	-7065.6134	7.3513	-12.7026
GPT-2_355M_	FFIx	1.0583	3.3161	0.0009	-7060.1057	-7065.6134	5.5077	-9.0154
GPT-2_774M_	FFIx	1.0820	3.1539	0.0016	-7060.6294	-7065.6134	4.9840	-7.9680
GPT-2_1.5B_	FFIx	1.1130	3.2304	0.0013	-7060.3857	-7065.6134	5.2277	-8.4553
GPT-Neo_125M_	FFIx	1.1728	3.4638	0.0005	-7059.6063	-7065.6134	6.0071	-10.0141
GPT-Neo_1.3B_	FFIx	1.0506	3.3282	0.0009	-7060.0657	-7065.6134	5.5476	-9.0953
GPT-Neo_2.7B_	FFIx	1.0218	3.0885	0.0020	-7060.8334	-7065.6134	4.7800	-7.5601
cloze_p_	FFIx	-10.7293	-2.6066	0.0092	-7062.2056	-7065.6134	3.4078	-4.8156
cloze_s_	FFIx	2.3591	3.8709	0.0001	-7058.1188	-7065.6134	7.4946	-12.9893
rating	FFIx	-3.8257	-3.1443	0.0017	-7060.6597	-7065.6134	4.9537	-7.9074
bigram	Fpass	1.8462	3.0919	0.0020	-7495.7488	-7500.5393	4.7905	-7.5810
trigram	Fpass	1.5903	3.1810	0.0015	-7495.4696	-7500.5393	5.0697	-8.1394
tetragram	Fpass	1.6175	3.4433	0.0006	-7494.6028	-7500.5393	5.9365	-9.8731
PSG	Fpass	2.4846	3.9080	0.0001	-7492.9011	-7500.5393	7.6383	-13.2766
RNN	Fpass	1.9731	2.9651	0.0031	-7496.1325	-7500.5393	4.4069	-6.8138
GPT-2_124M_	Fpass	3.0184	6.4610	$< .0001$	-7479.8471	-7500.5393	20.6923	-39.3845
GPT-2_355M_	Fpass	2.4999	5.8687	$< .0001$	-7483.4256	-7500.5393	17.1138	-32.2276
GPT-2_774M_	Fpass	2.5080	5.4708	$< .0001$	-7485.6446	-7500.5393	14.8947	-27.7894
GPT-2_1.5B_	Fpass	2.5679	5.5787	$< .0001$	-7485.0577	-7500.5393	15.4817	-28.9633
GPT-Neo_125M_	Fpass	2.7545	6.0984	$< .0001$	-7482.0768	-7500.5393	18.4625	-34.9250
GPT-Neo_1.3B_	Fpass	2.3512	5.5737	$< .0001$	-7485.0851	-7500.5393	15.4542	-28.9085
GPT-Neo_2.7B_	Fpass	2.4358	5.5110	$< .0001$	-7485.4271	-7500.5393	15.1122	-28.2245
cloze_p_	Fpass	-19.9252	-3.6055	0.0003	-7494.0327	-7500.5393	6.5067	-11.0134
cloze_s_	Fpass	4.3486	5.3278	$< .0001$	-7486.4060	-7500.5393	14.1334	-26.2668
rating	Fpass	-6.9527	-4.2593	$< .0001$	-7491.4751	-7500.5393	9.0643	-16.1286
bigram	RightBound	2.9715	4.3190	$< .0001$	-7703.5183	-7712.8367	9.3184	-16.6369
trigram	RightBound	2.5088	4.3548	$< .0001$	-7703.3638	-7712.8367	9.4729	-16.9457
tetragram	RightBound	2.4869	4.5947	$< .0001$	-7702.2993	-7712.8367	10.5373	-19.0747
PSG	RightBound	3.4518	4.7083	$< .0001$	-7701.7758	-7712.8367	11.0609	-20.1218
RNN	RightBound	2.8296	3.6850	0.0002	-7706.0416	-7712.8367	6.7951	-11.5902
GPT-2_124M_	RightBound	3.6020	6.6772	$< .0001$	-7690.7580	-7712.8367	22.0787	-42.1575
GPT-2_355M_	RightBound	2.9778	6.0527	$< .0001$	-7694.6462	-7712.8367	18.1905	-34.3810
GPT-2_774M_	RightBound	3.0687	5.7988	$< .0001$	-7696.1235	-7712.8367	16.7132	-31.4264
GPT-2_1.5B_	RightBound	3.1045	5.8412	$< .0001$	-7695.8811	-7712.8367	16.9556	-31.9112
GPT-Neo_125M_	RightBound	3.3894	6.5035	$< .0001$	-7691.8758	-7712.8367	20.9609	-39.9218
GPT-Neo_1.3B_	RightBound	2.8038	5.7545	$< .0001$	-7696.3747	-7712.8367	16.4620	-30.9240
GPT-Neo_2.7B_	RightBound	2.8913	5.6629	$< .0001$	-7696.8891	-7712.8367	15.9475	-29.8951
cloze_p_	RightBound	-22.5333	-3.5271	0.0004	-7706.6088	-7712.8367	6.2279	-10.4558
cloze_s_	RightBound	4.9069	5.1989	$< .0001$	-7699.3726	-7712.8367	13.4641	-24.9282
rating	RightBound	-8.7481	-4.6420	$< .0001$	-7702.0826	-7712.8367	10.7541	-19.5081
bigram	GoPast	5.0016	4.4580	$< .0001$	-8420.3914	-8430.3154	9.9239	-17.8479
trigram	GoPast	3.9280	4.1774	$< .0001$	-8421.5941	-8430.3154	8.7213	-15.4425
tetragram	GoPast	3.9031	4.4178	$< .0001$	-8420.5682	-8430.3154	9.7471	-17.4943
PSG	GoPast	5.3099	4.4360	$< .0001$	-8420.4885	-8430.3154	9.8268	-17.6537
RNN	GoPast	5.1277	4.0978	$< .0001$	-8421.9216	-8430.3154	8.3938	-14.7876
GPT-2_124M_	GoPast	3.7861	4.2642	$< .0001$	-8421.2303	-8430.3154	9.0851	-16.1702
GPT-2_355M_	GoPast	2.9394	3.6336	0.0003	-8423.7077	-8430.3154	6.6077	-11.2154
GPT-2_774M_	GoPast	3.1251	3.5946	0.0003	-8423.8478	-8430.3154	6.4676	-10.9351
GPT-2_1.5B_	GoPast	3.2206	3.6888	0.0002	-8423.5062	-8430.3154	6.8092	-11.6184
GPT-Neo_125M_	GoPast	3.8111	4.4487	$< .0001$	-8420.4323	-8430.3154	9.8830	-17.7661
GPT-Neo_1.3B_	GoPast	2.9638	3.7045	0.0002	-8423.4484	-8430.3154	6.8670	-11.7340
GPT-Neo_2.7B_	GoPast	2.9579	3.5278	0.0004	-8424.0850	-8430.3154	6.2304	-10.4608
cloze_p_	GoPast	-11.4677	-1.0961	0.2732	-8429.7116	-8430.3154	0.6038	0.7924
cloze_s_	GoPast	4.4228	2.8541	0.0044	-8426.2314	-8430.3154	4.0840	-6.1680
rating	GoPast	-11.4683	-3.7206	0.0002	-8423.3889	-8430.3154	6.9265	-11.8529
bigram	SPR	1.1713	3.4501	0.0006	-6668.0520	-6674.0118	5.9599	-9.9197
trigram	SPR	0.9809	3.4502	0.0006	-6668.0514	-6674.0118	5.9604	-9.9208
tetragram	SPR	0.9269	3.4678	0.0005	-6667.991	-6674.0118	6.0208	-10.0417
PSG	SPR	0.9586	2.6421	0.0083	-6670.5107	-6674.0118	3.5011	-5.0023
RNN	SPR	1.2262	3.2402	0.0012	-6668.7524	-6674.0118	5.2594	-8.5188
GPT-2_124M_	SPR	0.6284	2.3350	0.0197	-6671.2758	-6674.0118	2.7360	-3.4719
GPT-2_355M_	SPR	0.7977	3.2646	0.0011	-6668.6733	-6674.0118	5.3385	-8.6770
GPT-2_774M_	SPR	0.7477	2.8451	0.0045	-6669.9534	-6674.0118	4.0584	-6.1168
GPT-2_1.5B_	SPR	0.8626	3.2705	0.0011	-6668.6539	-6674.0118	5.3579	-8.7158
GPT-Neo_125M_	SPR	0.9050	3.4917	0.0005	-6667.9080	-6674.0118	6.1038	-10.2076
GPT-Neo_1.3B_	SPR	0.6255	2.5845	0.0098	-6670.6614	-6674.0118	3.3504	-4.7008
GPT-Neo_2.7B_	SPR	0.7990	3.1550	0.0016	-6669.0245	-6674.0118	4.9873	-7.9746
cloze_p_	SPR	-7.2889	-2.3118	0.0209	-6671.3298	-6674.0118	2.6820	-3.3640
cloze_s_	SPR	0.8142	1.7380	0.0824	-6672.4948	-6674.0118	1.5170	-1.0341
rating	SPR	-4.1687	-4.4909	$< .0001$	-6663.9418	-6674.0118	10.0700	-18.1401
bigram	ELAN	0.0084	0.5214	0.6021	-2191.0887	-2191.2254	0.1367	1.7266
trigram	ELAN	-0.0006	-0.0432	0.9656	-2191.2245	-2191.2254	0.0009	1.9981
tetragram	ELAN	-0.0012	-0.0977	0.9222	-2191.2206	-2191.2254	0.0048	1.9904
PSG	ELAN	-0.0179	-1.0454	0.2960	-2190.6762	-2191.2254	0.5492	0.9016
RNN	ELAN	-0.0052	-0.2907	0.7713	-2191.183	-2191.2254	0.0425	1.9151
GPT-2_124M_	ELAN	-0.0287	-2.2719	0.0232	-2188.635	-2191.2254	2.5904	-3.1808
GPT-2_355M_	ELAN	-0.0076	-0.6577	0.5108	-2191.008	-2191.2254	0.2174	1.5652
GPT-2_774M_	ELAN	-0.0070	-0.5670	0.5708	-2191.0638	-2191.2254	0.1616	1.6768
GPT-2_1.5B_	ELAN	-0.0068	-0.5502	0.5823	-2191.0733	-2191.2254	0.1522	1.6957
GPT-Neo_125M_	ELAN	-0.0202	-1.6541	0.0983	-2189.8511	-2191.2254	1.3743	-0.7486
GPT-Neo_1.3B_	ELAN	-0.0088	-0.7689	0.4421	-2190.9282	-2191.2254	0.2972	1.4056
GPT-Neo_2.7B_	ELAN	-0.0094	-0.7884	0.4306	-2190.9130	-2191.2254	0.3124	1.3751
cloze_p_	ELAN	0.0356	0.2402	0.8102	-2191.1964	-2191.2254	0.0290	1.9420
cloze_s_	ELAN	0.0115	0.5224	0.6015	-2191.0882	-2191.2254	0.1372	1.7256
rating	ELAN	-0.0790	-1.8009	0.0719	-2189.5966	-2191.2254	1.6288	-1.2576
bigram	LAN	-0.0165	-1.0156	0.3100	-2210.6890	-2211.2074	0.5184	0.9632
trigram	LAN	-0.01500	-1.1019	0.2707	-2210.5972	-2211.2074	0.6102	0.7797
tetragram	LAN	-0.0149	-1.1694	0.2424	-2210.5202	-2211.2074	0.6872	0.6256
PSG	LAN	-0.0261	-1.5066	0.1321	-2210.0671	-2211.2074	1.1402	-0.2805
RNN	LAN	-0.0122	-0.6765	0.4988	-2210.9774	-2211.2074	0.2300	1.5399
GPT-2_124M_	LAN	-0.0343	-2.6802	0.0074	-2207.6047	-2211.2074	3.6027	-5.2054
GPT-2_355M_	LAN	-0.0283	-2.4233	0.0155	-2208.2611	-2211.2074	2.9463	-3.8925
GPT-2_774M_	LAN	-0.0383	-3.0588	0.0023	-2206.5185	-2211.2074	4.6889	-7.3778
GPT-2_1.5B_	LAN	-0.0398	-3.1646	0.0016	-2206.1898	-2211.2074	5.0176	-8.0352
GPT-Neo_125M_	LAN	-0.0361	-2.9180	0.0036	-2206.9390	-2211.2074	4.2684	-6.5368
GPT-Neo_1.3B_	LAN	-0.0334	-2.8969	0.0038	-2207.0004	-2211.2074	4.2069	-6.4139
GPT-Neo_2.7B_	LAN	-0.0351	-2.9098	0.0037	-2206.9630	-2211.2074	4.2443	-6.4887
cloze_p_	LAN	0.3206	2.1344	0.0330	-2208.9206	-2211.2074	2.2868	-2.5735
cloze_s_	LAN	-0.0685	-3.0771	0.0021	-2206.4624	-2211.2074	4.7450	-7.4899
rating	LAN	0.2018	4.5639	$< .0001$	-2200.8099	-2211.2074	10.3975	-18.7951
bigram	N400	-0.0592	-4.3930	$< .0001$	-1938.4048	-1948.0434	9.6386	-17.2773
trigram	N400	-0.0584	-5.1880	$< .0001$	-1934.6354	-1948.0434	13.4081	-24.8161
tetragram	N400	-0.0576	-5.4504	$< .0001$	-1933.2586	-1948.0434	14.7848	-27.5696
PSG	N400	-0.0710	-4.9432	$< .0001$	-1935.8604	-1948.0434	12.1831	-22.3661
RNN	N400	-0.0762	-5.0852	$< .0001$	-1935.1568	-1948.0434	12.8866	-23.7732
GPT-2_124M_	N400	-0.0740	-7.0080	$< .0001$	-1923.7595	-1948.0434	24.2839	-46.5678
GPT-2_355M_	N400	-0.0635	-6.6035	$< .0001$	-1926.4421	-1948.0434	21.6013	-41.2027
GPT-2_774M_	N400	-0.0733	-7.1091	$< .0001$	-1923.0656	-1948.0434	24.9778	-47.9557
GPT-2_1.5B_	N400	-0.0750	-7.2397	$< .0001$	-1922.1559	-1948.0434	25.8876	-49.7751
GPT-Neo_125M_	N400	-0.0720	-7.0694	$< .0001$	-1923.3390	-1948.0434	24.7045	-47.4089
GPT-Neo_1.3B_	N400	-0.0683	-7.1955	$< .0001$	-1922.4653	-1948.0434	25.5781	-49.1562
GPT-Neo_2.7B_	N400	-0.0679	-6.8182	$< .0001$	-1925.0369	-1948.0434	23.0065	-44.0130
cloze_p_	N400	0.7627	6.1438	$< .0001$	-1929.3080	-1948.0434	18.7354	-35.4708
cloze_s_	N400	-0.1194	-6.4856	$< .0001$	-1927.1955	-1948.0434	20.8479	-39.6958
rating	N400	0.3084	8.4921	$< .0001$	-1912.6561	-1948.0434	35.3873	-68.7747
bigram	EPNP	0.0091	0.8064	0.4201	-1683.1571	-1683.4839	0.3269	1.3463
trigram	EPNP	0.0066	0.6919	0.4891	-1683.2433	-1683.4839	0.2406	1.5187
tetragram	EPNP	0.0062	0.6944	0.4875	-1683.2415	-1683.4839	0.2424	1.5152
PSG	EPNP	0.0058	0.4780	0.6327	-1683.3690	-1683.4839	0.1149	1.7702
RNN	EPNP	0.0120	0.9540	0.3403	-1683.0265	-1683.4839	0.4574	1.0852
GPT-2_124M_	EPNP	-0.0197	-2.1986	0.0281	-1681.0578	-1683.4839	2.4261	-2.8522
GPT-2_355M_	EPNP	-0.0140	-1.7157	0.0864	-1682.0054	-1683.4839	1.4785	-0.9570
GPT-2_774M_	EPNP	-0.0203	-2.3258	0.0202	-1680.7694	-1683.4839	2.7146	-3.4291
GPT-2_1.5B_	EPNP	-0.0222	-2.5287	0.0116	-1680.2762	-1683.4839	3.2077	-4.4154
GPT-Neo_125M_	EPNP	-0.0230	-2.6602	0.0079	-1679.9348	-1683.4839	3.5491	-5.0983
GPT-Neo_1.3B_	EPNP	-0.0243	-3.0294	0.0025	-1678.8844	-1683.4839	4.5995	-7.1990
GPT-Neo_2.7B_	EPNP	-0.0251	-2.9765	0.0030	-1679.0432	-1683.4839	4.4407	-6.8814
cloze_p_	EPNP	-0.0608	-0.5792	0.5626	-1683.3153	-1683.4839	0.1686	1.6628
cloze_s_	EPNP	-0.0153	-0.9805	0.3270	-1683.0008	-1683.4839	0.4831	1.0337
rating	EPNP	0.0753	2.4283	0.0153	-1680.5254	-1683.4839	2.9586	-3.9171
bigram	P600	-0.0177	-1.4627	0.1438	-1781.1356	-1782.2104	1.0748	-0.1496
trigram	P600	-0.0157	-1.5486	0.1217	-1781.0056	-1782.2104	1.2047	-0.4095
tetragram	P600	-0.0177	-1.8554	0.0637	-1780.4817	-1782.2104	1.7287	-1.4574
PSG	P600	-0.0209	-1.6142	0.1067	-1780.9015	-1782.2104	1.3089	-0.6177
RNN	P600	-0.0226	-1.6769	0.0938	-1780.7980	-1782.2104	1.4123	-0.8247
GPT-2_124M_	P600	-0.0447	-4.6967	$< .0001$	-1771.2032	-1782.2104	11.0072	-20.0143
GPT-2_355M_	P600	-0.0459	-5.3105	$< .0001$	-1768.1675	-1782.2104	14.0428	-26.0856
GPT-2_774M_	P600	-0.0497	-5.3610	$< .0001$	-1767.9017	-1782.2104	14.3086	-26.6173
GPT-2_1.5B_	P600	-0.0478	-5.1295	$< .0001$	-1769.1002	-1782.2104	13.1102	-24.2203
GPT-Neo_125M_	P600	-0.0459	-5.0055	$< .0001$	-1769.7213	-1782.2104	12.4891	-22.9781
GPT-Neo_1.3B_	P600	-0.0478	-5.5979	$< .0001$	-1766.6233	-1782.2104	15.5871	-29.1742
GPT-Neo_2.7B_	P600	-0.0513	-5.7444	$< .0001$	-1765.8058	-1782.2104	16.4045	-30.8091
cloze_p_	P600	0.1757	1.5657	0.1176	-1780.9789	-1782.2104	1.2314	-0.4628
cloze_s_	P600	-0.0636	-3.8312	0.0001	-1774.8679	-1782.2104	7.3425	-12.685
rating	P600	0.1847	5.6200	$< .0001$	-1766.5009	-1782.2104	15.7095	-29.419
bigram	PNP	0.0082	0.6475	0.5174	-1841.2269	-1841.4376	0.2107	1.5786
trigram	PNP	0.0063	0.5962	0.5512	-1841.2590	-1841.4376	0.1787	1.6427
tetragram	PNP	0.0059	0.5923	0.5537	-1841.2613	-1841.4376	0.1764	1.6473
PSG	PNP	0.0030	0.2232	0.8234	-1841.4126	-1841.4376	0.0251	1.9499
RNN	PNP	0.0066	0.4700	0.6384	-1841.3266	-1841.4376	0.1110	1.7779
GPT-2_124M_	PNP	-0.0070	-0.6998	0.4842	-1841.1915	-1841.4376	0.2462	1.5077
GPT-2_355M_	PNP	-0.0039	-0.4300	0.6673	-1841.3447	-1841.4376	0.0929	1.8141
GPT-2_774M_	PNP	-0.0081	-0.8301	0.4066	-1841.0913	-1841.4376	0.3463	1.3074
GPT-2_1.5B_	PNP	-0.0077	-0.7878	0.4309	-1841.1256	-1841.4376	0.3120	1.3760
GPT-Neo_125M_	PNP	-0.0100	-1.0397	0.2987	-1840.8944	-1841.4376	0.5432	0.9135
GPT-Neo_1.3B_	PNP	-0.0120	-1.3392	0.1807	-1840.5365	-1841.4376	0.9012	0.1977
GPT-Neo_2.7B_	PNP	-0.0136	-1.4416	0.1496	-1840.3935	-1841.4376	1.0441	-0.0883
cloze_p_	PNP	-0.1769	-1.5139	0.1303	-1840.2862	-1841.4376	1.1514	-0.3028
cloze_s_	PNP	0.0055	0.3157	0.7523	-1841.3875	-1841.4376	0.0501	1.8998
rating	PNP	0.0487	1.4089	0.1591	-1840.4403	-1841.4376	0.9973	0.0054

The first two columns of the table report the independent and the dependent variable of interest, respectively; the following columns indicate the regression coefficient ($\hat{B}$), *t* value and associated *p*  value, *LogLik* of the complete model, *LogLik* of the baseline, their difference ($\Delta$ *LogLik*), and the AIC difference ($\Delta$ *AIC*)

## Appendix C: Non-linear effects of predictability on ERP responses

In the body of the article, we analyzed the impact of several wpms on EEG and behavioral data through linear regression. Our choice was supported by the observation that log-probabilities are linearly related to both fixation and self-paced reading times, as discussed in the Introduction. However, it is still not clear whether the linearity assumptions hold for EEG responses. In the present section, we analyze the ERP measures considered in this study with non-linear spline-based regression by employing generalized additive models (GAMs; Wood, 2011). The motivation of this section is not to fully characterize the functional form of the effects of predictability on EEG data, but rather to test whether the results of the comparison between models that we performed in our inferential analyses hold when we allow for predictability estimates to be non-linearly related with EEG responses.

The GAM models were fitted with the Python package pyGam; all the predictors were entered linearly except for the predictability estimates, which were modeled as spline terms with a penalty on their second derivative, which encourages the functions to be smoother. The $\lambda$ parameter, which controls the strength of the regularization penalty for the spline terms, was set with a grid search. The analyses were performed with 5-fold cross-validation, where (a) grid-search and training and (b) testing were performed on disjoint subsets of the data.

Our results are summarized in Fig. 7, and reported in detail in the online supplementary materials. Notably, the pattern of results when considering LAN, N400, and P600 are virtually identical with respect to what we reported with linear regression, as the rank correlations with our previous results are almost perfect (LAN: $\rho$ = 1; N400: $\rho$ = 0.9929; P600: $\rho$ = 0.9964). As in the case of the linear analyses performed with cross-validation, EPNP amplitudes were not accounted for by any wpm. These results thus show that the assumption of linearity did not have any impact on the results that we described in the article.

## Footnotes

1 Indeed, if participants generate their continuations by unbiasedly sampling from their subjective probability distribution and we ignore inter-participant variability, then cloze probabilities would correspond to subjective probabilities (Smith & Levy, 2011).

2 The exact description of the construct that participants are asked to evaluate in such rating tasks can vary across studies, as raters may be asked to evaluate how well a word fits into a sentence (e.g., Rayner et al., 2001) or how expected is each sentence ending (Szewczyk & Federmeier , 2022). In the present study, we opted for this second formulation, since it stresses the focus on predictability rather than phrasal fit.

3 In the context of information-theoretic approaches to language processing, this value is often referred to as the *surprisal* of a word ($surprisal(w_i) = - \log p(w_i | w_1, w_2 \ldots w_{i-1}$); Levy, 2008).

4 Our choice of employing linear regression as our main analytical method is motivated by the general consensus on the view that the functional form of the effects of log-probability on behavioral observables is linear (at least when computed from text-based models, as motivated in the Introduction). However, the shape of predictability effects on EEG responses has not garnered a comparable attention (but see Szewczyk & Federmeier, 2022), and the possibility of different linking functions between predictability and ERP responses must be considered. For the sake of comparability, we present here the analyses performed with linear regression models, but we also analyzed the EEG responses with non-linear spline-based regression through Generalized Additive Models (GAMs; Wood, 2011). The results of the non-linear analyses are reported in Appendix C.

5 We thank Stefan Frank and one anonymous reviewer for raising this concern.

6 Frequency is in fact a form of unigram probability, i.e., an *N*-gram model with context size equal to zero.

7 Note, however, that LAN effects emerge more clearly in a cross-validation setting if the predictability estimates are included non-linearly as predictors in the models (see Appendix C).

8 Note that this trend is reported only for relatively large transformer models; under a certain threshold – which is yet to be empirically established – larger models have an advantage over the smaller ones. For instance, Merkx & Frank (2021) report that a two-layer transformer outperforms a single-layer one in accounting for human reading data.

9 In this paragraph we always refer to the results obtained on eye movement data without spillover, as they allow to better discriminate among the different eye-tracking measures.

10 Note also that GoPast is a more noisy eye-tracking measure, as demonstrated by the fact that our baseline explains less variance in comparison with the other models ($LogLik_{baseline}$ = -8430.3154; FFix: $LogLik_{baseline}$ = -7065.6134; Fpass: $LogLik_{baseline}$ = -7500.5393; RightBound: $LogLik_{baseline}$ = -7712.8367).

11 Note that Frank et al. (2015) found a significant effect of predictability only on the N400 component; however, they only considered surprisal estimates derived from *N*-gram models, RNN, and PSG as independent variables. Figure 3 clearly shows that predictability effects on LAN, EPNP, and P600 only emerge if considering transformer- or human-based estimates.
